# The effect of incorporating different concentrations of chlorhexidine digluconate on the degree of conversion of an experimental adhesive resin

**DOI:** 10.4317/jced.54391

**Published:** 2018-04-01

**Authors:** Lamiaa-Mahmoud Moharam, Haidy-Nabil Salem, Hanaa-Mahmoud Elgamily

**Affiliations:** 1 Restorative and Dental Materials Department, National Research Centre, Giza, Egypt

## Abstract

**Background:**

The aim of this study was to evaluate the effect of chlorhexidine digluconate incorporation on the degree of conversion of an experimental adhesive resin.

**Material and Methods:**

The experimental resin was prepared from 70 wt% bisphenol A glycerolate dimethacrylate, 30 wt% hydroxyethyl methacrylate, silanized SiO2 nanofillers, 0.5% of camphorquinone and ethyl 4-dimethylaminebenzoate (binary photo-initiator system). Five chlorhexidine digluconate concentrations (0, 0.5, 1, 2 and 4 wt%) were then incorporated into the experimental resin. Thirty Potassium Bromide pellets were prepared then divided into six groups (n=5/group), repre¬senting the tested adhesive resins (Single Bond 2, 0, 0.5, 1, 2 and 4 wt% chlohexidine-incorporated experimental adhesive resins), that were applied to the pellets without light-curing (uncured specimens). Another 30 pellets were prepared and treated with the previous materials then light-cured using LED light-curing device (cured specimens). Degree of conversion of the uncured and the cured specimens were evaluated using FTIR analysis.

**Results:**

Adper Single Bond 2 showed the highest degree of conversion mean values followed by 0.5 wt% chlorhexidine concentration then 2 wt% followed by 4 wt% then 1 wt% concentrations, while 0 wt% concentration showed the lowest mean values.

**Conclusions:**

Chlorhexidine digluconate had slight significant influence on the efficiency of polymerization of the experimental adhesive resin.

** Key words:**Chlorhexidine digluconate, different concentrations, degree of conversion, experimental adhesive resin.

## Introduction

Dentin bonding agents are intermediate materials that endorse adhesion between the resin composite materials and the tooth. Nonetheless; the success of bonding to the dental hard tissues represents a major challenge for adhesive dentistry (1). Optimal monomer infiltration into the previously demineralized collagen network and the accomplishment of higher degrees of resin monomer conversion are considered critical factors for establishing a long-lasting resin/dentin bonding. The degree of conversion (DC) of resinous materials is the degree to which the carbon double bonds C=C are altered into carbon single bonds C-C (2). The degree of conversion of dental resins is a major factor which greatly affects the ultimate physical and mechanical properties of such resins. The degree of conversion and cross-linking density are heavily influenced by several factors such as the chemical structure of the monomer, the wavelength and intensity of the light curing devices, as well as the addition of different anti-bacterial agents in order to eliminate the harmful effect of the bacteria and their by-products which negatively affect the bond quality and the tooth structure itself (3).

Chlorhexidine (CHX) is a broad spectrum antibacterial agent which is frequently applied to the prepared cavity walls prior to permanent restoration placement. Carrilho *et al.* (4) suggested that CHX inhibits the action of the matrix metalloproteinase enzymes (MMPs). MMPs are proteolytic enzymes, which are produced by the partially demineralized dentin. These enzymes can be activated by contemporary self-etch and etch-and-rinse adhesives. MMPs have the capability to hydrolyze the exposed collagen fibrils located at the bottom of the hybrid layer due to failure of the adhesive resin to infiltrate to the full depth of the partially demineralizd dentin leading to reduced bond strength as well as reduced bond durability (5). Many manufacturers recommend the use of different disinfectants prior to etch-and-rinse adhesives applications (6). This might be attributed to the acid etching step which is responsible for the removal of the smear layer formed on the cut dentin surface during cavity preparation. Removal of the smear layer might enable CHX to easily infiltrate into the full depth of the underlying demineralized dentin, so that the CHX has greater chance to deactivate the MMPs. Moreover, some studies showed that applying CHX disinfectants directly on etched dentin surfaces would be a better alternative than applying it on the unetched dentin surfaces that are covered with smear layer (7).

In spite of the beneficial findings of the use of 2% CHX, as a non-rinse primer on etched dentin, this procedure added an extra step to the bonding protocol, which is against the clinicians’ preference for simplification. On the other hand, other studies evaluated the impact of CHX incorporation in the acid conditioner or in the adhesive solution. The CHX inclusion in the phosphoric acid was found to be capable of preventing the resin/dentin bond degradation after six months of water storage (8). Controversial results have been published concerning CHX inclusion in dental primers and/or adhesives, this could be attributed to the variations in the CHX concentration and the type of the bonding strategy evaluated (9). However, before testing the effect of CHX-incorporated adhesive resins on the durability of resin/dentin bond, it would be more logical to inspect whether the incorporation of CHX into dental adhesives modiﬁes their polymerization, and hence, affecting their ﬁnal degree of conversion and the quality of polymerization of the adhesive resin.

Therefore, this study was undertaken to evaluate the degree of conversion of a nano-filled methacrylate-based experimental adhesive resin after incorporation of CHX digluconate in five different concentrations.

## Material and Methods

One commercially available etch-and-rinse adhesive resin (Adper Single Bond 2, 3M ESPE Dental Products, St. Paul, MN, USA), and an experimental light-cured, nano-filled, dimethacrylate-based adhesive resin were used in this study. CHX digluconate was added to the experimental adhesive resin in five different concentrations (0 wt%, 0.5 wt%, 1 wt%, 2 wt% and 4 wt%). Materials, compositions, descriptions and manufacturers are presented in  Table 1.

**Table 1:**
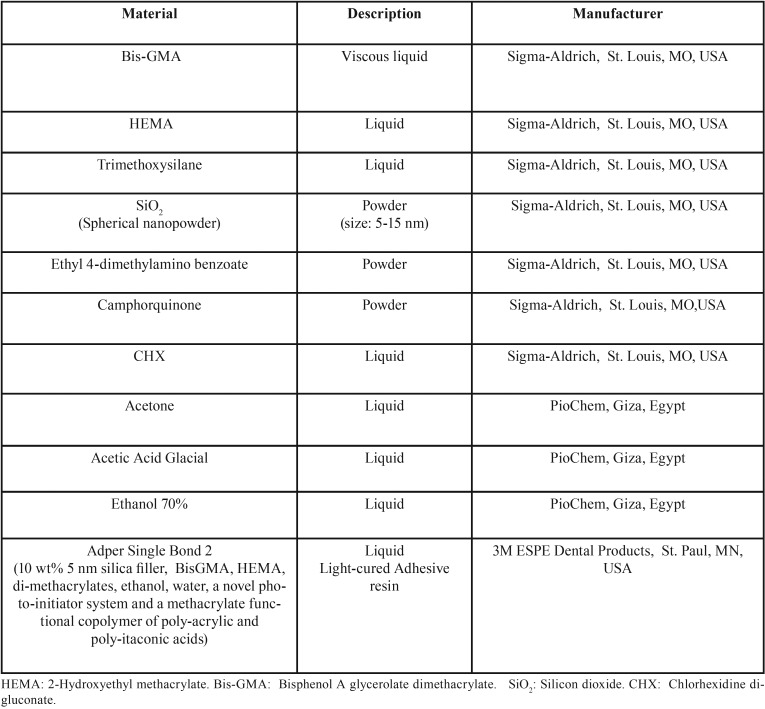
Materials, composition, description and manufacturer.

-Preparation of the experimental adhesive resin:

The monomer was prepared by mixing 70 wt% bisphenol A glycerolate dimethacrylate (Bis-GMA) and 30 wt% hydroxyethyl methacrylate monomers (HEMA). A binary light-curing system, composed of 0.5% camphorquinone and 0.5% ethyl 4-dimethylamine benzoate, was dissolved in the mixture to make the mixture light curable (10). Different components of the resin matrix were weighed using a digital sensitive balance (AE ADAM, PW124 Lab Balance, Adam Equipment Co Ltd, Kingston, MK, UK). In order to dissolve the adhesive in acetone and ethanol, a solvent volume equivalent to 10 wt% of acetone and 10 wt% of ethanol was added to the mixture. The mixture was continuously stirred for two h using a small magnet on a magnetic stirrer (Wisestir MSH-300, Witeg Labortechnik, Wertheim, Germany) to ensure homogenization of all components as well as dissolution of the monomers into the solvents (11). To increase the hydrolysis rate of the silane coupling agent, a few drops of acetic acid were gradually added to 40 ml of 70% ethanol solution in order to decrease the pH to 3˗4. Three wt% of the silane coupling agent was added to the pH-adjusted solution, and the solution was stirred for one h. Then the SiO2 nanoparticles were immersed directly into the prepared solution. The mixture was centrifuged for 30 min. The remaining ethanol was removed and the precipitate was dried using air pressure oven at 105 °C (12). A concentration of 0.1 wt% of silanized SiO2 nanoparticles was added to the experimentally prepared adhesive resin. The mixture was stirred in the magnetic stirrer for two h till complete dispersion of the nanoparticles (13). CHX digluconate solution in concentrations of 0.5, 1, 2 and 4 wt%, respectively, were added to the previous experimental mixture.

**Figure 1:**
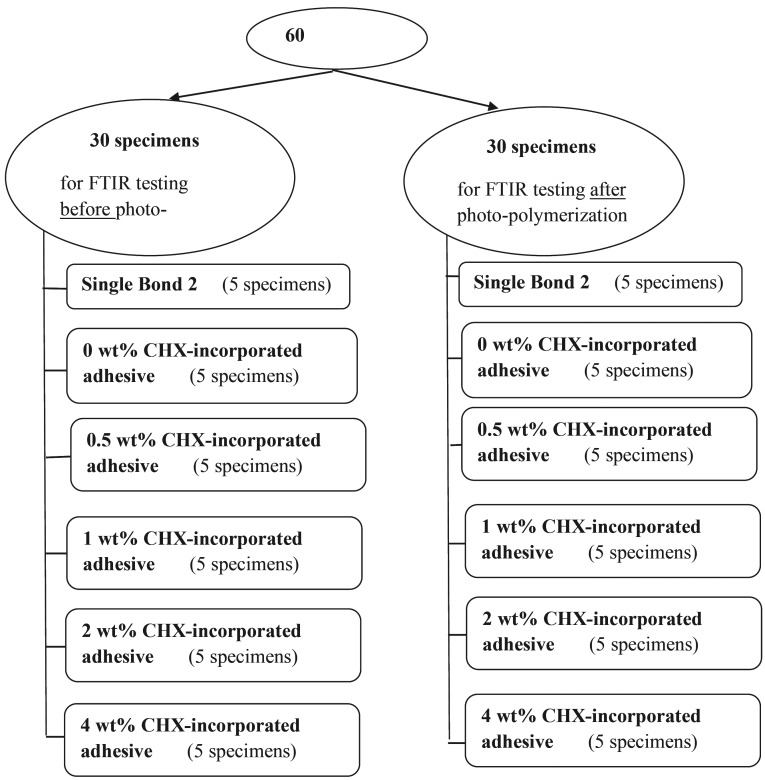
Equation.

-Study design and experimental grouping:

In the current study, Fourier transform infrared spectroscopy (FTIR) test was conducted using Potassium Bromide (KBr) pellet technique in order to evaluate the DC of the tested adhesive resins (14). FTIR is a technique which is used to acquire an infrared spectrum of absorption or emission for the different tested materials. KBr is the most commonly used alkali halide in pellets preparation, which does not show any absorption spectrum in the infrared region inside the FTIR different devices. Moreover, it does not interact with the different tested materials. Sixty (KBr) pellets were prepared and used throughout the whole study. Thirty KBr pellets were equally divided into six groups, (n=5/group), representing the different tested adhesive resins [Adper Single Bond 2 adhesive resin & 0, 0.5, 1, 2 and 4 wt% CHX-incorporated experimental dental adhesive resins]. The tested adhesives were applied to the top surface of the pellets without light-curing (photo-polymerization), to represent the uncured (un-polymerized) control specimens. Another 30 KBr pellets were divided into six groups, (n=5/group), representing the different adhesives applied to the top surface of the pellets same as before, and then light-cured (photo-polymerized) using the light curing unit, to represent the cured (photo-polymerized) test specimens. LED (light emitting diode) light curing unit (Elipar S10, 3M ESPE Dental Products, St. Paul, MN, USA) was used with an output of 1000 mW/cm2. The output of the light curing unit was periodically checked using a hand-held radiometer (Demetron 100, Kerr Corporation, Orange, CA, USA), (Fig. 1).

-Degree of Conversion (DC%):

Specimens’ preparation and application of the adhesive resins:

Nine mg of Ispectropic grade (IR) of KBr powder was placed in a special specimen holder, and then it was pressed to form a 1 mm thickness transparent pellet under heavy pressure for one min, using a pellet maker kit (KBr Product-A-Press, International Crystal Labs, Garfield, NJ, USA). (14) All tested adhesive resins (Adper Single Bond 2 adhesive resin, 0, 0.5, 1, 2 and 4 wt% CHX-incorporated experimental adhesive resins) in the current study were applied to the top surfaces of the prepared KBr pellets according to the commercial adhesive resin manufacturer’s instructions. For the un-cured specimens (control specimens); one drop of each of the tested adhesive resins was dispensed into a mixing well. Two separate coats of each adhesive were applied to the top surface of the KBr pellet using a disposable micro-brush with gentle agitation for 15 s. Then each adhesive resin was air-dried using gentle compressed oil-free air for five s. The pellets were then evaluated immediately for their DC.

For the light-cured specimens (test specimens); each adhesive resin was applied the same way as before, then light-cured as close as possible from the top surface of the KBr pellets for 10 s using the LED light curing unit. The pellets were then stored in light-proof containers for 24 h before evaluation of the DC.

**Figure 2:**
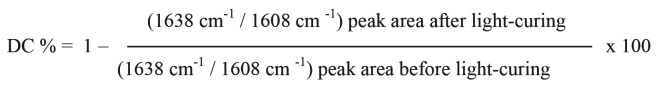
Flow chart of the specimens grouping in the current paper.

-Degree of Conversion (DC %) testing:

FTIR spectra of the un-cured (control) and light-cured adhesive resin specimens were obtained using 24 scans at 4 cm–1 in the absorbance mode (Jasco FT-IR 6400, JASCO International Co. Ltd, Tokyo, Japan), using the KBr pellet technique (14). For calculating the degree of conversion DC%, the percentage of unreacted carbon-carbon double bonds (% C=C) was determined from the ratio of absorbance peak areas of aliphatic carbon-carbon double bonds C=C (peak at 1638 cm-1) against aromatic component C-C (peak at 1608 cm–1) which was used as an internal standard before and after light-curing (photo-polymerization). The underlying peak area was calculated for each peak, using a standard baseline technique (15) with the aid of a computer software program provided with the spectrometer (Spectra Manager Version 2, JASCO, Umstadt, Germany). The degree of monomer conversion was determined using the following equation: (Fig. 2).

-Statistical Analysis

Numerical data were explored for normality by checking the data distribution and using Kolmogorov-Smirnov and Shapiro-Wilk tests. All data showed parametric distribution. Data were represented as mean, standard deviation (SD), median, range and 95% Confidence interval (95% CI) value. One-way ANOVA test was used to compare between the six groups. Tukey’s post-hoc test was used for pair-wise comparisons when ANOVA test was significant. The significance level was set at *P* ≤ 0.05. Statistical analysis was performed with SPSS (IBM Corporation, NY, USA) Statistics Version 20 for Windows.

## Results

One-way ANOVA test in  Table 2 showed that there was a statistically significant difference between the groups at *P*<0.001.

**Table 2:**
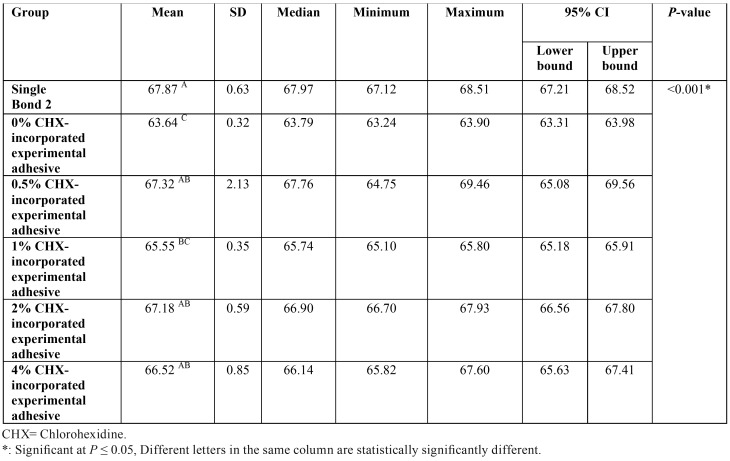
One-way ANOVA for comparison between the degree of conversion values (%) of the different tested adhesive resins.

Pair-wise comparisons between the groups revealed that Single Bond 2 showed the highest mean degree of conversion (67.87 ± 0.63) with no statistically significant difference with 0.5%, 2% and 4% CHX-incorporated experimental adhesive resin groups (67.32±2.13, 67.18±0.59 and 66.52±0.85 respectively). A lower mean degree of conversion was recorded for 1% CHX-incorporated experimental adhesive resin group (65.55±0.35). It showed a statistically significantly lower mean value than Single Bond 2 group (67.87 ± 0.63) but no statistically significant difference with 0 wt% CHX-incorporated experimental adhesive resin groups (63.64±0.32). The statistically significantly lowest mean degree of conversion was obtained with 0% CHX-incorporated experimental adhesive resin group (63.64±0.32), ( Table 2).

## Discussion

In the present study, the experimental adhesive resin was prepared with a traditional ratio 70:30 of Bis-GMA/HEMA which was used in formulation of experimental dental resin adhesives in previous studies (12,13). Bis-GMA is a high molecular weight monomer, responsible for the rigid properties of the resin matrix. Low molecular weight monomer HEMA constituted the remaining 30%, in order to allow for full resin penetration into etched dentin. A concentration of 0.1 wt% of silanized SiO2 nanoparticles was incorporated into the experimental adhesive resin, to mimic the filler loading in the commercial adhesive resin, and at the same time it assured high amount of low molecular weight monomers. The nano-fillers were silanized with silane coupling agent, which is based on 3-methacryloxypropyltrimethoxy silane that is able of bonding via its alkoxysilane groups with the filler particles, and with the resin because of its methacrylate functional group (16).

Dental adhesive systems still encounter some clinical problems, particularly related to the imperfect infiltration of the resin monomers into the demineralized dentin surface during the bonding step as well as the reduction in the degrees of monomer conversion upon photo-polymerization (4). The incorporation of resin monomers with CHX (0.2˗2 wt% concentrations) was found to increases the bond strength and preserves the durability of dental adhesives (17), however; The inclusion of CHX in primers and/or adhesives has been an issue of debate and many controversial results have been published which could be attributed to variations in the amount of CHX concentration and the type of bonding strategy evaluated (9,18). Using dental adhesive resins as vehicles for the delivery of different and effective therapeutic agents, in order to improve the durability of the resin/dentin adhesive bond, represents an important target for many researchers and manufacturers (19). In the current study, a signiﬁcant decrease in DC mean values was observed when CHX was not impregnated into the experimental adhesive resin (0 wt% CHX concentration in the experimental adhesive resin followed by 1 wt% CHX concentration). On the other hand; CHX incorporation into the experimental adhesive resin had increased the DC at concentrations of 4 wt%, 2 wt % and 0.5 wt% groups respectively without statistical significant difference between such groups.

On the other hand, Adper Single Bond 2 adhesive showed the highest DC mean value while the lowest mean value was recorded for 0 wt% CHX˗incorporated experimental adhesive resin groups. This might be attributed to the different concentrations of the nano˗silica filler particles in both adhesives, which was 10 wt% in case of Adper Single Bond 2 compared to 0.1 wt% of nano˗silica filler particles in case of the 0 wt% CHX˗incorporated experimental adhesive resin group. Increasing the nano˗filler loading within the adhesive systems might have a direct positive effect on the quality as well as degree of polymerization of the tested different adhesive resins, which intern might have increased the degree of monomer conversion into the final polymer. Almost all commercial etch-and-rinse adhesive systems include HEMA in their composition (13,20) to improve the infiltration of hydrophobic monomer into the demineralized dentin, leading to enhanced micro˗mechanical retention of the photo-polymerized monomers. Unfortunately, water sorption and polymer degradation over time can be encouraged due to presence of such hydrophilic components in the hybrid layer (21), whereas HEMA raises adhesive layer permeability, and diminishing the mechanical properties of the hybrid layer by time. The influence of HEMA on mechanical properties of polymer structure might be attributed to the low degree of conversion exhibited by polymers containing increased concentration of HEMA. A low degree of conversion is also related to a low crosslink density and decreased mechanical properties of the finally formed polymer (20). Consequently, the previous findings agreed with the results of the present study, in which Adper Single Bond 2 adhesive resin showed the highest DC mean values in comparison to the other experimental tested groups. As Adper Single Bond 2 adhesive, contains only 5˗15% HEMA while the experimentally prepared adhesive resin contained 30% HEMA. In addition, Adper Single Bond 2 adhesive resin contains a new photo˗initiator system that might have more free radicals generated during the photo˗polymerization process leading to faster monomer conversion and higher values of its final DC. On the other hand; the tested experimental adhesive resin contains camphorquinone which represents the traditional photo˗initiator system in dental adhesive resins. Camphorquinone might have less free radicals generated during photo˗polymerization leading to lower DC values (22). In the present study, among the CHX˗impregnated experimental adhesive resin groups; the 0.5 wt% CHX concentration showed the highest non˗significant DC values followed by 2 wt% then 4 wt%, while the lowest DC mean values were recorded for the 1 wt% CHX˗incorporated adhesive resin group. This could be attributed to the incomplete polymerization of the polymer because of the presence of CHX drug particles (23). By increasing the concentration of the CHX into the adhesive, this action will consequently decrease the ability of the resin to convert monomers into polymers upon polymerization and hence, the final DC will be decreased. Cadenaro *et al.* (19) agreed with the results of this study. They concluded that; increasing the concentrations of CHX dissolved in different dental adhesive resin blends had a little adverse effect on DC. Furthermore, CHX had slight significant influenced the efficiency of polymerization compared to the positive control group (0 wt% CHX˗incorporated experimental adhesive) probably due to protonation of CHX (24). Moreover; the shift in the peak corresponding to the carbonyl group in the impregnated test groups compared to the positive control group could be attributed to hydrogen/bonding interactions with N˗H groups of CHX molecules. This finding was in accordance with a previous study which concluded the presence of a shift in carbonyl group in CHX˗impregnated specimens compared to the non˗impregnated specimens (25). This *in vitro* study revealed clinically relevant results, as they represented the efficiency of incorporation of an antibacterial agent such as CHX digluconate into the experimentally prepared adhesive resin. Such incorporation was shown to have a significant effect on the final DC and hence the quality of the polymerization of the adhesive resin which is highly desired in the clinical situation.

Last but not least, more studies are needed to evaluate other properties of adhesive resins with CHX impregnation. Thus, the optimal concentration of CHX that might be added in the adhesive to produce stable bonds without jeopardizing other mechanical properties of the adhesive layer is yet to be addressed.

## Conclusions

Under the limitations of the current study; adding chlorhexidine digluconate to the experimentally prepared adhesive resin had the potential to increase the degree of monomer conversion of the adhesive resin. Incorporation of chlorhexidine digluconate with 4 wt%, 2 wt% and 0.5 wt% concentrations into the experimentally prepared adhesive resin showed a comparable and promising effect on the degree of monomer conversion upon comparison with Adper Single Bond 2.
